# Activation of the hypoxia response in the aging cerebrovasculature protects males against cognitive impairment

**DOI:** 10.1111/acel.14264

**Published:** 2024-07-02

**Authors:** Peihu Li, Xiaoman Bi, Dahua Xu, Yuan Meng, Yucheng Xia, Jiale Cai, Yutong Shen, Jiaqi Wang, Jiazhu Chen, Lamei Yin, Bo Wang, Deng Wu, Kongning Li

**Affiliations:** ^1^ College of Biomedical Information and Engineering Hainan General Hospital, Hainan Affiliated Hospital of Hainan Medical University Haikou China; ^2^ Wannan Medical College Wuhu China; ^3^ School of Life Sciences, Faculty of Science The Chinese University of Hong Kong Hong Kong China

**Keywords:** Alzheimer's disease, blood brain barrier, hypoxia, sex, vascular aging

## Abstract

Alzheimer's disease (AD) is a neurodegenerative disorder with a distinct sex bias. Age‐related vascular alterations, a hallmark of AD onset and progression, are consistently associated with sexual dimorphism. Here, we conducted an integrative meta‐analysis of 335,803 single‐nucleus transcriptomes and 667 bulk transcriptomes from the vascular system in AD and normal aging to address the underlying sex‐dependent vascular aging in AD. All vascular cell types in male AD patients exhibited an activated hypoxia response and downstream signaling pathways including angiogenesis. The female AD vasculature is characterized by increased antigen presentation and decreased angiogenesis. We further confirmed that these sex‐biased alterations in the cerebral vascular emerged and were primarily determined in the early stages of AD. Sex‐stratified analysis of normal vascular aging revealed that angiogenesis and various stress‐response genes were downregulated concurrently with female aging. Conversely, the hypoxia response increased steadily in males upon aging. An investigation of upstream driver transcription factors (TFs) revealed that altered communication between estrogen receptor alpha (*ESR1*) and hypoxia induced factors during menopause contributes to the inhibition of angiogenesis during normal female vascular aging. Additionally, inhibition of *CREB1*, a TF that targets estrogen, is also related to female AD. Overall, our study revealed a distinct cerebral vascular profile in females and males, and revealed novel targets for precision medicine therapy for AD.

## Graphical abstract

Cerebrovasculature aging leads to blood–brain barrier damage, contributing to the onset of Alzheimer's disease (AD). The male brain produces significant levels of hypoxia‐inducible factors that stimulate angiogenesis and safeguard brain tissue. Increased antigen presentation and decreased angiogenesis capacity during the cerebrovasculature aging in females contribute to a higher risk of developing AD.

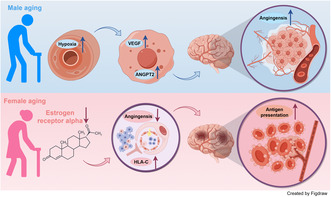

**Abbreviations**
AD: Alzheimer's disease
BBB: blood–brain barrier
BSA: bovine serum albumin
DEGs: differentially expressed genes
EadDEG: early‐stage related adDEGs
Endo: endothelial cells
Epd: ependymal cells
ESR1: estrogen receptor alpha
FAD: familial Alzheimer's disease
Fib: fibroblasts
GRNs: gene regulatory networks
LRT: likelihood ratio test
ORA: overrepresentation analysis
PC: principal component
Per: pericytes
SMCs: smooth muscle cells
snRNA‐seq: single‐nucleus RNA sequencing
TFs: transcription factors
VageGenes: vascular normal aging genes

## 1 INTRODUCTION

Alzheimer's disease (AD), a neurodegenerative disorder caused by damage to neurons in the brain, is the most common cause of dementia, accounting for 60%–80% of dementia cases (Julie et al., 2007). According to the World Alzheimer Report 2015 (*Alzheimer's & Dementia*, 2023), 46.8 million individuals are currently living with dementia worldwide. This number is expected to increase to approximately 131.5 million by 2050, which will place an increasing burden on families and society. Approximately two‐thirds of AD patients are females (Castro‐Aldrete et al., 2023). Moreover, the lifetime risk of developing AD is greater in females than in males. The lifetime risk of developing AD at age 45 is approximately 20% for females and 10% for males (Nebel et al., 2018). Furthermore, after being diagnosed with AD, females also experience cognitive impairment at a quicker pace. Although sex differences are notable, prior studies have not considered the effect of sex or have only adjusted for sex as a covariate (Castro‐Aldrete et al., 2023; Nebel et al., 2018), leading to imprecise parsing of the pathologic characteristics of AD.

The two primary pathological hallmarks of the AD brain are senile plaques, which are formed by the deposition of Aβ, and neurofibrillary tangles, which are caused by hyperphosphorylated tau. These issues have been the focus of drug development for decades (Zhang et al., 2021). Nevertheless, pharmacotherapies targeting Aβ and tau cannot prevent the gradual loss of neurons or impede progressive cognitive decline in AD patients (Godyń et al., 2016). A probable reason for this is that therapies are often initiated during the latter stages of the disease, when significant neural damage has already occurred. Therefore, there is a crucial need to redirect research toward the early stages of the disease. The blood brain barrier (BBB), a protective layer composed of endothelial cells with tight junctions, maintains brain homeostasis and its disruption is an early feature of AD (Sweeney et al., 2019). The BBB is harmed by vascular aging, which interferes with proper nutrient delivery, waste elimination, vascular perfusion, and immunological function. Recently, published articles have indicated that accelerated artery aging is associated with the early development of AD (Oh et al., 2023). On the other hand, there are also variations in vascular aging between sexes (Ji et al., 2022). Compared to that in males, the vascular system in females expresses more mineralocorticoid receptors (DuPont et al., 2021) and is less sensitive to the baroreflex. An aging‐induced decrease in estrogen also causes endothelial dysfunction in the vasculature of middle‐aged and older females (Moreau et al., 2012). Older and postmenopausal females are more susceptible to vascular lesions as a result of these variations, and vascular abnormalities are one of the risk factors for the onset of AD (Arvanitakis et al., 2016).

There has been a significant amount of single‐cell RNA research on AD brain tissue (Jiang et al., 2020). Researchers have identified cells associated with AD in various brain regions and at different stages of the disease, investigated the dysfunction of diverse cell types in the AD brain, and analyzed AD‐specific changes in transcriptome profiles. However, there has been a lack of focus on sex‐specific differential changes in AD. Additionally, there is a notable scarcity of cerebrovascular data for AD patients, which is even more challenging to obtain from healthy individuals. To explore the causes of the sex‐biased pathogenesis of AD from the perspective of the vascular aging and provide innovative ideas for sex‐specific treatment in AD, an in‐depth investigation of human brain endothelial transcriptome at single‐nuclei resolution is urgently needed.

Here, we showed that male AD patients had increased angiogenesis and hypoxia responses in their cerebral blood vessels. In contrast, female AD patients exhibited impaired vascular growth and development. Moreover, these sex‐specific pathogenic differences were evident in the early stages of the disease. Additionally, we employed data from the Genotype‐Tissue Expression (GTEx) project to identify genes associated with vascular aging in both healthy males and females, and revealed that normal vascular aging may contribute to the observed sex differences in AD. We further investigated sex‐specific transcription factors (TFs) that could regulate these disparities. Ultimately, our study suggests potential pathways for sex‐specific therapeutic interventions.

## 2 MATERIALS AND METHODS

### 2.1 Human cerebrovascular snRNA‐seq data from Na Sun

In this study, we utilized data from Sun's research (Sun et al., 2023) involving a total of 428 normal and AD patients of both sexes, encompassing 22,514 cells. We processed the generated cell‐gene count matrices using Seurat (v.4.3.0.1) and carried out a quality control step, filtering out cells with ≤200 genes, ≥5000 unique molecular identifiers, ≥5% mitochondrial genes, or ≥5% ribosomal protein genes. Subsequently, we identified the top 2000 highly variable genes for dimension reduction using the Seurat default parameters. These highly variable genes were subjected to principal component (PC) analysis, and the top 30 PCs were selected for nonlinear dimension reduction (UMAP). Before proceeding to clustering, we performed batch effect correction using Harmony and estimated doublet scores using DoubletFinder (v2.0.3), assuming a doublet formation rate of 20%. Doublet cells were excluded from further analysis. We then performed clustering with a resolution setting of 0.5 and visualized the data using UMAP and cell type labels from the original study.

### 2.2 Identification of DEGs between individuals with AD (including those early AD) and controls

We compared differentially expressed genes (DEGs) between individuals with AD (including early AD) and controls. We applied MAST (Model‐based Analysis of Single‐cell Transcriptomics), a single‐cell‐based method, to measure the statistical significance of each gene based on a linear model. The covariates, including the number of cells, the number of expressed genes, race, PMI, age, and batch, were controlled in the model. Genes with *p* < 0.05 and an absolute log2 fold change (|log2FC|) greater than 0.1 were selected as AD DEGs (adDEGs) (early‐adDEGs) for further analysis.

### 2.3 Overrepresentation analysis

We utilized the Metascape tool, and selected human species for overrepresentation analysis (ORA) of all pathway sets using default parameters (Min Overlap ≥3, *p* < 0.01 as the cutoff). The genes selected for male–female differential term analysis were all the genes included in the subpathways. The R package UCell (v2.4.0) was used to score the male–female differential pathways.

### 2.4 Validation with independent datasets

We began our analysis by acquiring the dataset from Lau et al. (2020) (GSE157827). This dataset, generated via single‐nucleus RNA sequencing (snRNA‐seq) and comprising a total of 169,496 nuclei, encompasses all cell types within the prefrontal cortex of the brain. This allowed us to investigate the expression of AD GWAS risk genes across a diverse range of cell types. We conducted an extensive quality control process, eliminating cells with ≤200 genes or ≥2000 unique molecular identifiers. Subsequently, we identified the top 1000 highly variable genes for dimension reduction using the default parameters of Seurat. We then selected the top 20 PCs for nonlinear dimension reduction via UMAP. Clustering was performed with a resolution setting of 1, and data visualization was carried out using UMAP, incorporating cell type labels from the original study. Within this dataset, we employed the EWCE on all GWAS loci for AD genes that we collected from PubMed (Table S12), to scrutinize their expression patterns across different cell types. We extracted the data for endothelial cells. Using the R package presto (v1.0.0), we applied the wilcoxauc function to the processed Seurat object to calculate the differences between male and female AD patients. We then sorted the genes by their log2FC from high to low, using this as the background gene set for GSEA. We then validated the sex‐adDEGs using clusterProfiler (v4.8.3) for GSEA, and GseaVis (v0.0.9) for result visualization. This dataset was also used to verify the expression of *PIK3C2A*.

Additionally, we downloaded the dataset from Yang et al. (2022) (GSE163577). This dataset was created using a vessel enrichment protocol to concentrate brain vascular cells in brain tissue samples from individuals with AD and controls and comprises a total of 143,793 single‐nucleus transcriptomes. From this dataset, we extracted data pertaining to endothelial cells from the prefrontal cortical area of AD patients of different sexes. For quality control, we removed outliers with a high ratio of mitochondria (more than 5%, fewer than 200 features) relative to endogenous RNAs and homotypic doublets (more than 5000 features) using Seurat. Following sctransform normalization and integration, we filtered out doublets and multiplets using DoubletFinder. We also used cell markers from the original study to label the UMAP clusters. We validated sex‐differing pathway genes using the same GSEA method as described above. This dataset was also used to verify the expression of individual genes.

### 2.5 Mouse 5XFAD

C57BL/6J mice typically coexpress five familial Alzheimer's disease (FAD) mutations, which are known to accelerate the development of amyloid pathology. All mice were maintained in a specific pathogen‐free environment at a temperature of 21–23°C, with humidity levels between 40% and 60%, and a 12‐h light/12‐h dark cycle. At 9 months of age, the mice were euthanized by overdose anesthesia before tissue collection.

### 2.6 IHC staining

Brain tissues were collected from the mice and fixed in 10% neutral‐buffered formalin for 24 h. Following fixation, the tissues were cryoprotected by sequential immersion in 10%, 20%, and 30% sucrose solutions in PBS at 4°C until they sank. Tissues were then embedded in optimal cutting temperature compound and frozen in isopentane cooled with dry ice. Sections 10–15 μm thick were cut using a cryostat and mounted on positively charged slides. The sections were air‐dried for 30 min at room temperature.

For immunohistochemistry, the sections were rehydrated in PBS and permeabilized with 0.3% Triton X‐100 in PBS for 15 min. Nonspecific binding was blocked by incubating the sections with 5% bovine serum albumin (BSA) in PBS for 30 min. The sections were then incubated overnight at 4°C with the primary antibody (1:100 dilution) in PBS containing 1% BSA (anti‐HIF1 alpha: 340462, China; anti‐Angiopoietin 2: RM5425, China). After washing in PBS, the sections were incubated with a biotinylated secondary antibody for 30 min at room temperature, followed by incubation with a streptavidin‐HRP conjugate. The signal was developed using DAB substrate, and the sections were counterstained with hematoxylin. Finally, the sections were dehydrated, cleared in xylene, and mounted with permanent mounting medium. The stained sections were examined and imaged using a light microscope.

### 2.7 Baseline comparison of differential pathway genes between sexes

To investigate the differences between males and females in the expression of genes involved in AD pathways within the context of normal vasculature, we first collected RNA sequencing data of the aorta and tibial artery from different age groups of both sexes in normal individuals from the GTEx database (GTEx Consortium, 2013). We initially processed the data using the variance‐stabilizing transformation function in the DESeq2 (v1.38.3) package, followed by the removal of batch effects using the function provided in the limma package. Subsequently, we utilized the Wilcoxon test to determine the differences in the expression of genes within sex‐differentiated pathways in normal males and females across various age groups, setting a significance threshold at *p* ≤ 0.05 We then analyzed the genes whose expression differed according to sex at each stage and determined their correlation (Spearman) with the median age.

### 2.8 Identification of a gene set associated with vascular aging

The vascular aging genes were identified by analyzing the aorta and tibial artery data from the GTEx database, which included all complete age stages, using DESeq2 (v1.38.3) and the likelihood ratio test (LRT). The LRT included tissue and the Hardy scale to eliminate their potential impact on aging. The significance threshold for age‐related genes was set at an adjusted *p* ≤ 0.05. The significant age‐related genes were scaled using the remove batch effect function in the limma (v3.54.2) package, with tissue and Hardy score set as covariates. The scaled gene expression matrix was used as input for the degPatterns function in DEGreport (v1.34.0) to perform clustering based on gene expression pattern similarity. Clusters of genes with progressive and consistent trends were manually selected as common aging genes.

### 2.9 Transcription factor analysis

The single‐cell rEgulatory network inference and clustering (PySCENIC) tool was utilized to infer TF activities and gene regulatory networks (GRNs) from single‐cell RNA‐seq data. Initially, we processed single‐cell RNA‐seq data from the prefrontal cortex, which were scaled to 1000 samples, and saved them in loom format, making them compatible with PySCENIC's requirements for subsequent processing. The gene expression matrix and the list of human TFs were used as inputs for the GRNBoost2 algorithm to infer the GRN. Next, we used the resulting regulon matrix and the downloaded hg38 human genome annotation file for CisTarget analysis, and finally, the AUCell tool was used for scoring TF activity. The SCENIC package (v1.3.1) in R was then used to retrieve information about the TFs. We selected TFs that controlled at least two target genes and used Cytoscape (v3.7.2) to display their interactions. The Wilcoxon test was used to compare TF changes between patients with AD and normal controls.

### 2.10 Docking of molecules

We searched for the structures of three compounds, namely, rolipram, catechins, and ginsenoside, in PubChem and downloaded them in SDF format. These structures were then converted into mol2 format using PyMOL (v2.5.7). We downloaded the structure of the CREB1 protein from the AlphaFold Protein Structure Database. Molecular docking was performed using AutoDockTools (v4.2.6) (Morris et al., 2009). The results of the molecular docking were visualized in PyMOL.

## 3 RESULTS

### 3.1 Endothelial cell‐enriched genes in the prefrontal cortex show a sex‐biased gene expression pattern

**FIGURE 1:**
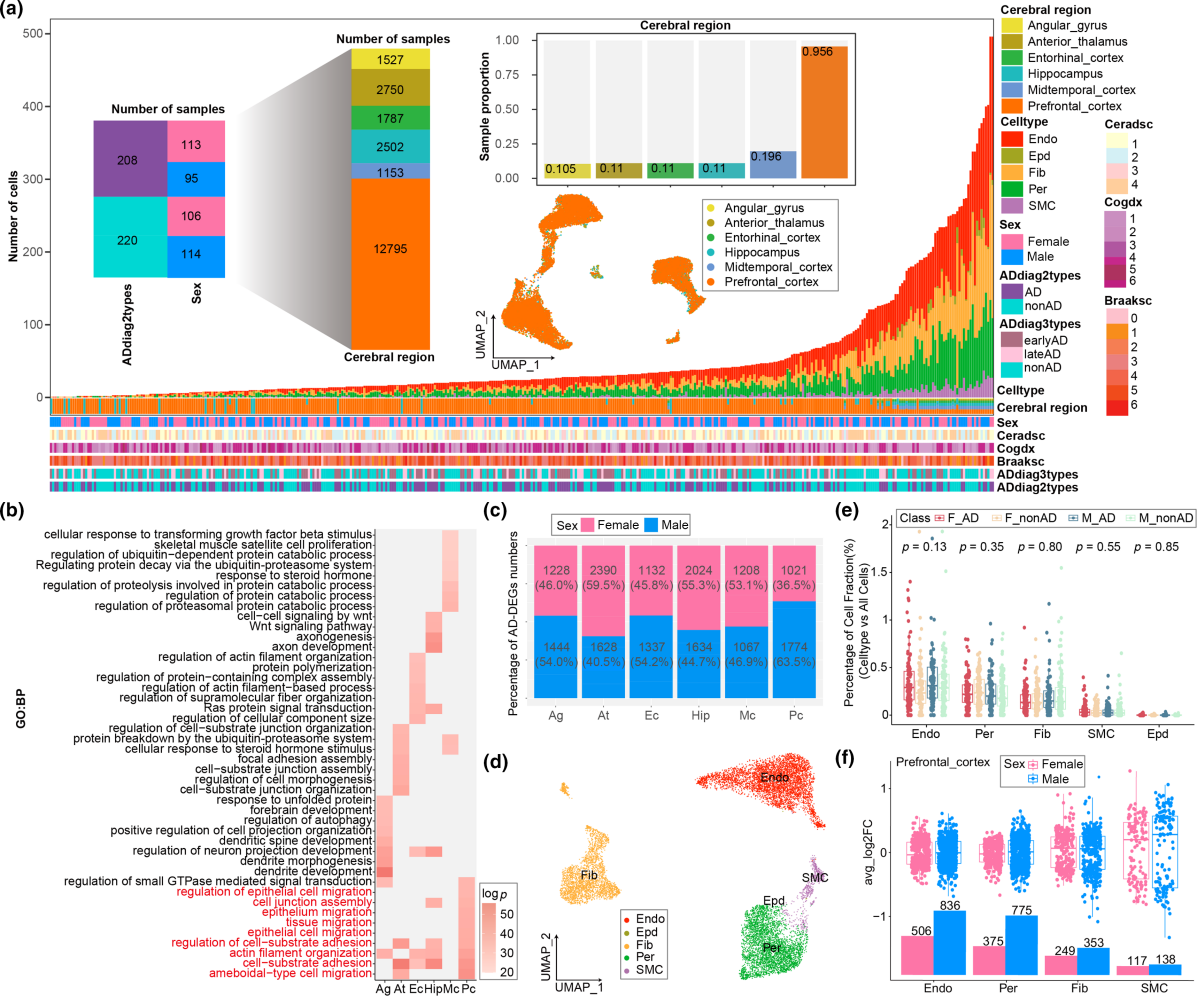
Analysis of adDEGs in different brain regions and cell types. (a) Sample information statistics. (b) Biological process overrepresentation analysis of adDEGs in different brain regions. (c) Ratio of male to female adDEGs in different brain regions. (d) UMAP plot of single cells from the prefrontal cortex. (e) Boxplot of the proportions of different cell types in males and females with and without alzheimer's disease (AD) in the prefrontal cortex relative to all cells. (f) Distribution of the number of adDEGs and log2‐fold change (log2FC) in different cells in the prefrontal cortex in males and females with AD.

Previous studies performed in neuroanatomy have highlighted the various subcortical areas that play distinct roles in sexual response and cognitive impairment (Cosgrove et al., 2007). To better understand their sex‐bias in AD progression in the single‐nuclei transcriptome, a pilot meta‐analysis of a single‐nuclei RNA sequencing (snRNA‐seq) dataset from Sun et al. (2023), consisting of sex‐ and age‐matched samples from 428 individuals across six brain areas (Figure 1A; Table S1) was performed. In total, five cell types including endothelial cells (Endo), smooth muscle cells (SMCs), ependymal cells (Epd), pericytes (Per), and fibroblasts (Fib), were identified from a total of 22,514 nuclei (Figure 1A) using their established markers (Figure S1A). Sex‐stratified vascular cell type proportion comparisons across six regions (Figure S1B) revealed similar proportions across six brain regions, and no sex‐disproportion types were observed.

To further investigate the effect of brain region‐specific vascular disturbance on AD pathology progression, we compared gene expression between AD patients and normal individuals. Biological process ORA revealed that the adDEGs from the prefrontal cortex participated in several essential vascular processes, including cell adhesion, cell junction assembly, and cell migration (Vestweber, 2008), rather than the neuronally supported functions enriched by adDEGs from other regions (Figure 1B). The prefrontal cortex showed a sex‐biased adDEG pattern that was dominated by males. (Figure 1C). Based on these findings and the knowledge of cognitive impairment‐related brain regions, we hypothesized the vascular dysfunction in the prefrontal cortex is more strongly attributed to female during AD progression.

To gain insight into the cell type‐specific contribution to the dysregulation of the prefrontal cortex, we concentrated on the prefrontal cortex nuclei and identified five major cell types (Figure 1D). Among these, endothelial cells had the highest proportion (Figure 1E) and displayed the greatest number of adDEGs in both females and males (Figure 1F). Additionally, there was a modest increase in the number of endothelial cells in AD patients, which is consistent with their denser blood vessels (Fernandez‐Klett et al., 2020). Furthermore, we found that AD‐related risk genes, such as *IGHG1*, *IGHG3*, *FOXF1*, and *ADAMTS1*, which were previously known to be highly expressed in microglia, are markedly expressed in endothelial cells (Figure S1C), further highlighting the role of endothelial cells in AD. Taking these results together, the dysregulation of endothelial cells in the prefrontal cortex may contribute to the sex bias of AD patients. In the following analysis, we focused on the variation in endothelial cells.

### 3.2 Divergent hypoxia response and antigen presentation determine sex discrepancies in AD

**FIGURE 2:**
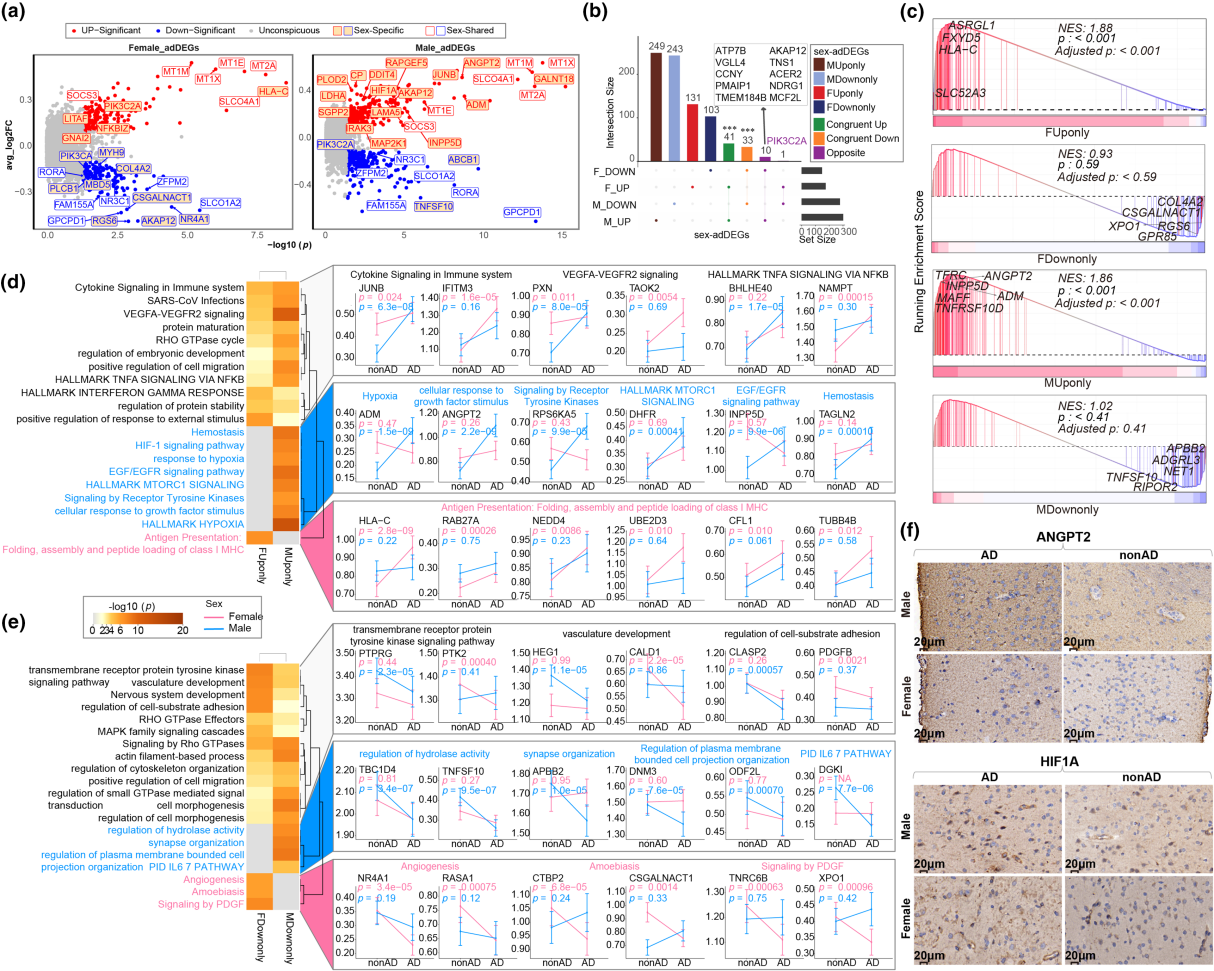
Differential analysis of Alzheimer's disease (AD) between endothelial cells in the prefrontal cortex of different sexes. (a) Volcano plot displaying adDEGs in brain vascular endothelial cells for both males and females. (b) UpSet plot showing the distribution of upregulated and downregulated DEGs in brain vascular endothelial cells in both sexes (MUponly/MDownonly: Male‐specific up/down‐regulated adDEGs; FUponly/FDownonly: Female‐specific up/down‐regulated adDEGs; Congruent Up/Down: AdDEGs up/down‐regulated in both sexes; Opposite: AdDEGs regulated in opposite directions between sexes). (c) The validation of male and female sex‐adDEGs in the single‐cell data of prefrontal cortex endothelial cells from Shun‐Fat Lau's study using GSEA. The x‐axis is sorted from high to low based on AD log2FC values. (d–e) The Metascape tool was used for overrepresentation analysis of sex‐adDEGs in brain vascular endothelial cells (left), and the expression patterns of the most significant genes in important pathways are shown, displaying the mean gene expression and its 95% confidence interval (right) (duplicate genes are skipped). The MAST test was used to determine *p*‐values. (f) Immunohistochemistry results showing increased ANGPT2 and HIF1A expression in the cerebrovasculature of male AD mice.

Overall, 319 and 577 adDEGs were identified in the endothelial cells of the female‐AD and male‐AD prefrontal cortices, respectively, compared to those in the normal cortex (Tables S2 and S3). Among these genes, 41 upregulated and 33 downregulated genes were found to be shared by both sexes (Table S4). Among the commonly upregulated genes were members of the metallothionein gene family involved in neuroprotection, including *MT1M*, *MT1X*, *MT1E*, and *MT2A*, and sodium‐independent organic anion transporters including *SLOC4A1* and *SLOC1A2*. The commonly downregulated genes, such as *GPCPD1* and *NR3C1*, are involved in lipid metabolism (Figure 2A). These limited variations in shared genes suggest that ion transport and lipid metabolism are dysregulated in AD patients regardless of sex. As expected, a significant overlap was found between male and female adDEGs (Figure 2B). The 41 adDEGs that were upregulated in both male and female AD patients indicated a common hypoxic and increased immune response, whereas the 33 adDEGs that were codownregulated revealed a reduction in vascular functions, such as lipid and heme metabolism (Figure S2A).

Notably, the majority of the adDEGs (90.88%) displayed sex‐specific variation or even opposite directionality between females and males (Figure 2A,B; Table S4). For instance, *HLA‐C*, an MHC class I heavy chain receptor, showed the most significant increase in female patients with AD, but did not change in male patients with AD (Figure 2A). In contrast, *TNFSF10*, a ligand that induces apoptosis, was significantly downregulated in males but not in females (Figure 2A). In total, we obtained 131/249 sex‐specific upregulated genes and 103/243 downregulated genes in females and males, respectively. We validated the sex‐specific gene list with an independent dataset that contained 21 samples (Lau et al., 2020), even though our discovery cohort represented the largest AD‐associated snRNA‐seq dataset at the time. The results were comparable (Figure 2C). In particular, the top up‐ and downregulated sex‐specific genes, such as *HLC‐C* and *TNFSF10*, were thoroughly verified with their corresponding patterns (Figure 2C).

ORA revealed specific activation of the hypoxia response signaling‐centric network in male AD patients (Figure 2D). Additionally, signaling pathways closely related to or downstream of this network, including the EGF/EGFR signaling pathway, receptor tyrosine kinase signaling pathway, mTOR signaling pathway, and VEGFA signaling pathway, were significantly increased in male patients with AD (Figure 2D). This finding was further confirmed by an independent dataset (Figure S2B,C). Additionally, hypoxia activation was exclusively observed in pericytes and fibroblasts from male AD patients (Figure S3), suggesting that the hypoxia response is a common signaling pathway activated in all vascular types of the male AD patients. Accordingly, activities related to angiogenesis, one target of the hypoxia signaling pathway, were significantly increased in males (Figure 2D; Table S5). We also focused on a few genes in these pathways (*ADM*, *RAPGEF5*, and *ANGPT2*) due to their established functions in angiogenesis, neuroprotection, and cellular processes (Chrzanowska‐Wodnicka, 2010; Fernandez et al., 2016; Lv et al., 2022). We next validated the robustness of these male‐enriched pathways and genes using the Yang dataset (Yang et al., 2022). The results revealed that male‐specific pathways, including the hypoxia response, EGFR, and growth factor signaling pathways, were enriched in upregulated genes in male AD patients and downregulated in females (Figure S2B,C). Similarly, the abovementioned *ADM*, *ANGPT2*, and *RAPGEF5* genes also exhibited expression differences between male and female AD patients, further supporting our findings (Figure S2D). Additionally, we used an AD mouse model for immunohistochemistry experiments, which confirmed our findings. The results showed increased expression of angiogenesis (Angiopoietin‐2, ANGPT2) and hypoxia‐inducible (hypoxia inducible factor 1 subunit alpha, HIF1A) genes in the cerebrovasculature of male AD mice (Figure 2F). Distinct from males, female AD patients exhibited specific activation of an antigen presentation‐associated process in female AD (Figure 2D), which is consistent with the specific increase in the *HLA‐C* gene. Considering the activation of immune response pathways including the interferon response, TNFA signaling pathway, and cytokine signaling pathways, we postulated that there is a severe degree of endothelial dysfunction in female patients with AD. Furthermore, the specific inhibition of angiogenesis in female patients with AD (Figure 2E; Table S6) further confirmed our speculation. We also observed this inhibition in both pericytes and smooth muscle cells in females (Figure S3).

In addition to the sex‐specific alternated genes described above, we identified 11 directionally opposite genes between female and male AD patients (Figure 2B). Among these genes, 10 genes decreased in females and increased in males, and were significantly enriched in the DNA damage response signaling pathway (Figure S2E), a pathway that prevents cells from undergoing apoptosis, suggesting activated endothelial cell survival protection in male AD. In contrast, only *PIK3C2A*, a gene from the phosphoinositide 3‐kinase family, significantly increased in female AD but decreased in male AD. Previous studies validated that activation of *PIK3C2A* stimulates the activation of vascular endothelial growth factor and then promotes angiogenesis, indicating that female AD possesses its own fixed mechanism to achieve the same angiogenesis level as male AD. The parallel outcome of *PIK3C2A* was observed in two other independent snRNAseq datasets (Lau et al., 2020; Yang et al., 2022) (Figure S2F) further validating the crucial role of *PIK3C2A* in female AD and providing a potential druggable target for male AD.

### 3.3 Sex‐specific endothelial alterations were initiated and detected during early‐stage AD

**FIGURE 3:**
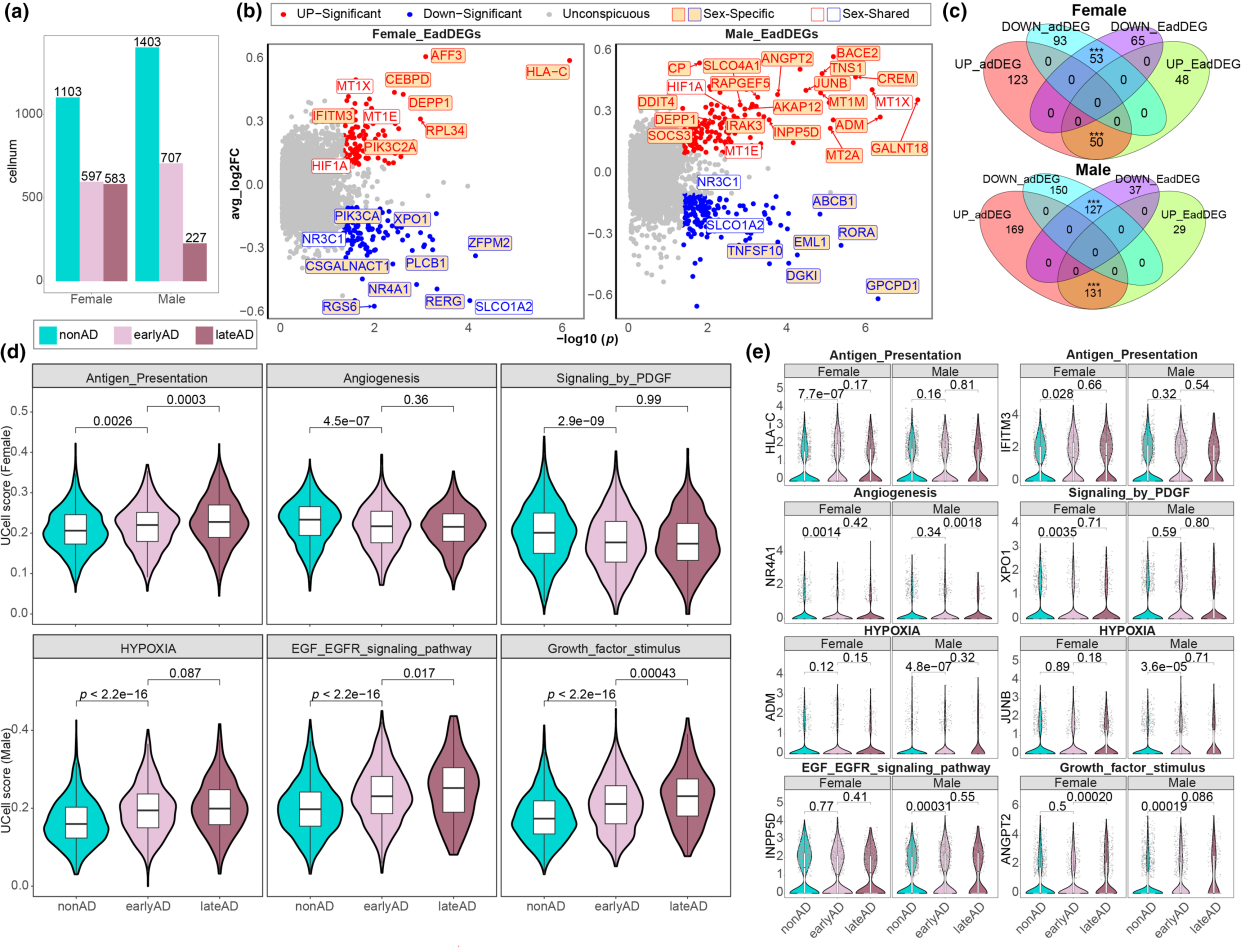
An illustration of sex differences in Alzheimer's disease (AD) at an early stage. (a) Bar plot showing the distribution of early and late‐stage samples in both males and females. (b) Volcano plots of early‐adDEGs in female (left) and male (right) brain vascular endothelial cells. (c) Venn diagrams showing overlap between early‐adDEGs and full‐stage adDEGs in females (top) and males (bottom) *** indicates a hypergeometric test with *p* < 0.001. (d) Ucell score plots of differential pathways at different AD stages for females (top) and males (bottom) (Wilcox test). (e) Violin plots showing the expression of key genes in differential pathways at different stages of AD in both sexes (MAST test).

The above results highlighted the activation of the hypoxia response and antigen representation characterized by sex‐specific endothelial gene expression during AD progression. In an attempt to examine whether these divergent responses initiated from early‐stage AD pathology, three subgroups corresponding to the pathological progression of AD were obtained from Sun et al. (2023) based on six clinicopathological traits together with cognitive impairment (Figure S4). In agreement with the more severe pathology observed in females with AD than in males with AD, a significantly greater number of females were diagnosed with late‐stage AD (Figure 3A).

Gene expression perturbations during non‐AD pathology versus early‐stage AD pathology were quantified, and 216 and 324 early‐stage related adDEGs (EadDEG) were identified in females and males, respectively (Figure 3B). A total of 32.29% of the adDEGs in females (53 downregulated EadDEG/146 adDEGs, and 50 upregulated EadDEG/173 adDEGs) and 44.71% of the adDEGs in males (127 downregulated EadDEG/277 adDEGs, and 131 upregulated EadDEG/300 adDEGs) were recognized as EadDEG genes with a directionally concordant pattern in their corresponding sex (Figure 3C). Notably, the top commonly upregulated genes, including *MT1X*, and the sex‐specific regulated genes, including increased *HLA‐C* in females, *ADM* in males, and decreased *TNFSF10* in males, exhibited the most significant changes in early‐stage AD, with the same trend occurring in the corresponding sex.

We next evaluated the variation in the whole sex‐specific pathways during early‐stage AD using normalized U statistics by treating the genes from the corresponding pathway as a single signature. As expected, hypoxia response‐ and antigen representation‐associated signatures significantly increased in males and females during early‐stage AD (Figure 3D). Intriguingly, the antigen representation‐associated signature continued to increase significantly in late‐stage AD patients, while the hypoxia response‐associated signature did not significantly change between late‐ and early‐stage AD patients, suggesting that gene expression perturbations in AD patients occur at the early‐stage. Furthermore, hypoxia‐related pathways, including the EGF/EGFR signaling pathway and growth factor‐stimulated cellular response, significantly increased in the early‐stage of male AD and continuously increased in the late stage. Hypoxia downstream processes, including angiogenesis and PDGF signaling, were significantly decreased in the early‐stage of female AD but did not change between late‐stage and early‐stage AD. Correspondingly, the most significantly altered genes related to these processes and pathways were significantly altered between early‐stage and non‐AD but not between late‐stage and early‐stage AD (Figure 3E). Overall, we propose that sex‐specific endothelial alterations are initiated and largely determined at the early‐stage of AD progression.

### 3.4 Vascular normal aging predicts sex differences in AD

**FIGURE 4:**
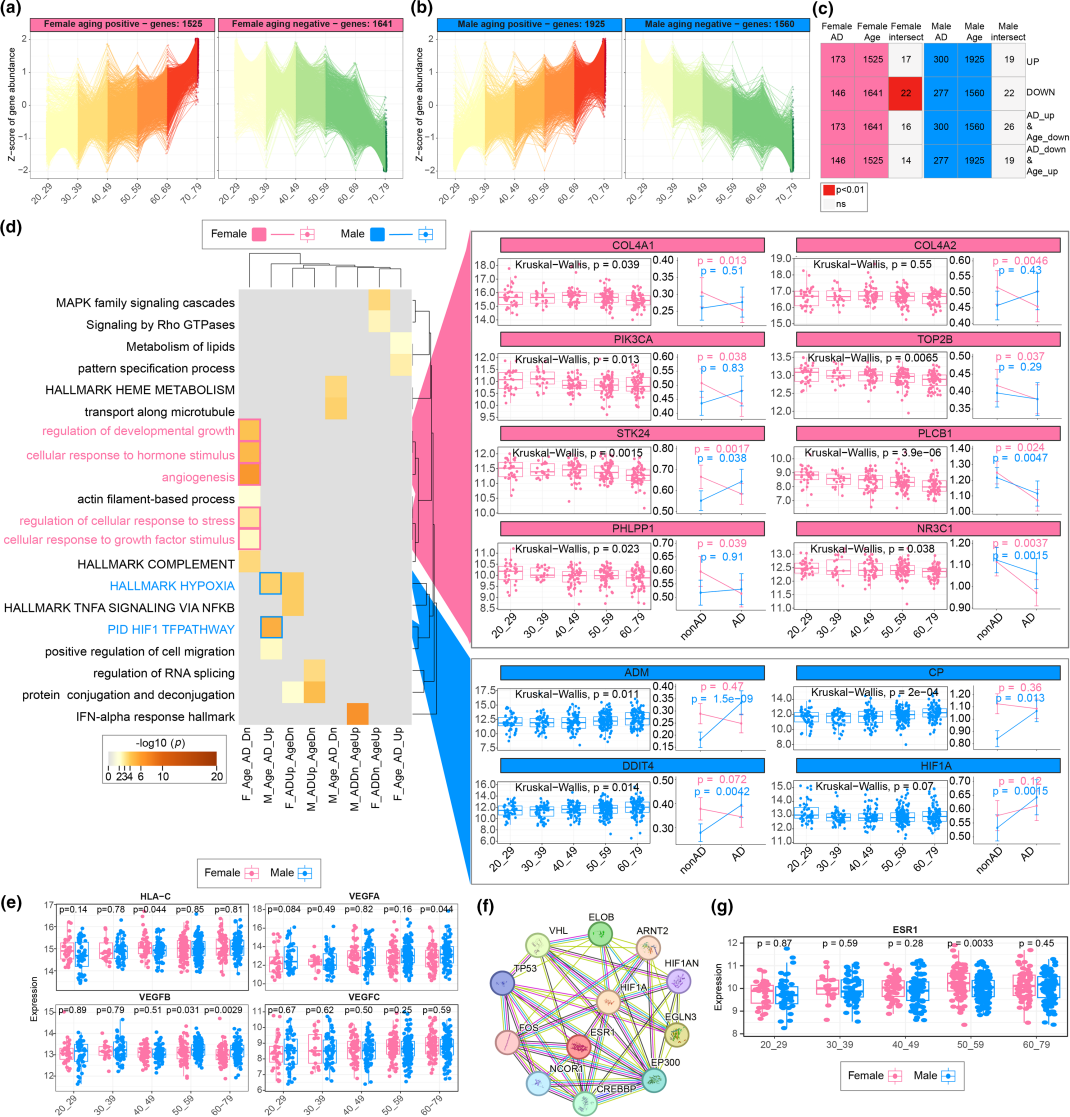
Sex differences in patients with Alzheimer's disease (AD) resulting from normal vascular aging. (a, b) DegPattern clustering graphs of VageGenes in normal vessels for both females (a) and males (b). (c) Overlap diagram of adDEGs and VageGenes in males and females (hypergeometric test). (d) Metascape overrepresentation analysis chart of vascular aging AD differential genes (left) and alterations in key pathway genes (right). (e) Aging status of HLA‐C and VEGF family genes. (f) Estrogen receptor ESR1 and hypoxia‐inducible factor HIF1A protein–protein interaction network map. (g) Changes in ESR1 during normal vascular aging in males and females.

Aging is a key risk factor for AD, and vascular aging creates lesions that alter vascular growth and development capacity, the hypoxic response, and antigen presentation pathways (Burtscher et al., 2021), consistent with our observations of sex‐specific endothelial variation in AD. Considering that gene expression perturbations occur during the progression of early‐stage AD, a subgroup closely related to the non‐AD pathology, we speculated that there is potential crosstalk between normal vascular aging and the initiation of AD pathology. To validate this hypothesis, healthy blood vessel transcriptome profiles from 667 healthy individuals (female: *n* = 250, male: *n* = 417) aged between 20 and 79 years were collected from the GTEx database to identify normal vascular aging associated genes. In total, 4889/4035 aging‐associated DEGs (female/male) were obtained and divided into 44/26 groups (female/male) based on their scaled gene expression with age (Figures S5 and S6), and the groups whose expression continuously increased or decreased with increasing age were selected as the vascular normal aging genes (VageGenes) (Figure 4A,B). In total, 3166 (1525 aging‐positive and 1641 aging‐negative) and 3485 (1925 aging‐positive and 1560 aging‐negative) VageGenes were acquired from females and males, respectively (Table S7). The VageGenes we described have considerable overlap with the aging datasets, and they also have significant overlap between sexes (Figure S7A,B). Pathway enrichment analysis of different VageGenes between males and females revealed that both sexes exhibit common aging features. These features included increased immune response and adhesion factors, as well as decreased cellular functions, such as cytolytic metabolism, cell division, and cell cycle regulation (Figure S7C). The dysregulation of these functions aligns with the known characteristics of endothelial cell aging (Jia et al., 2019). Moreover, when the enrichment pathways are the same, the degree of significance is different, with a stronger immune response in normal male vascular aging and a more severe decline in cellular function in females.

Direct comparison between the adDEGs and VageGenes revealed that 15.07% (22/146) of the aging negatively related female VageGenes were significantly downregulated in female AD patients (Figure 4C; Table S8). The ORAs revealed that angiogenesis (*COL4A1*, *COL4A2*, and *PIK3CA*) and several cellular responses to stress (*TOP2B*, *STK24*, *PLCB1*, and *PHLPP1*), hormones (*NR3C1*, *PIK3CA*, and *PLCB1*), and growth factors (*COL4A2*, *NR3C1*, and *PIK3CA*) significantly decreased with age and further decreased in females with AD (Figure 4D; Table S9). The most specific highly expressed gene in female patients with AD, *HLA‐C*, displayed a continuous trend with normal aging (Figure 4E). Intriguingly, the expression of hypoxia response process‐associated genes (*ADM*, *CP*, *DDIT4*, and *HIF1A*), which were repeatedly observed to be specifically increased in male AD patients, significantly increased with normal vascular aging in males (Figure 4D). Additionally, the levels of vascular growth factors, targets of hypoxia signaling and regulators of angiogenesis, including *VEGFA*, *VEGFB*, and *VEGFC*, continuously increased with normal vascular aging (Figure 4E). Overall, gene expression in AD patients is significantly affected via normal aging in both females and males.

The above findings collectively highlighted the activation of the hypoxia stress response and its downstream pathways, including angiogenesis, in males during normal aging and AD progression. However, why is hypoxia signaling inhibited in females during normal aging (Figure 4D)? Considering the functional equivalence between estrogen and hypoxia (George et al., 2012) and that common targets between estrogen receptor‐α (Erα) and hypoxia‐inducible factor 1α (HIF‐1α) as well as Erα directly regulate the expression of *HIF1A* (Yang et al., 2015), we speculated that the dysregulation of Erα, which is caused by variations in estrogen hormones during normal aging in females, disturbed the hypoxia response. To validate this hypothesis, we evaluated the correlation between *ESR1* and *HIF1A* using the String database and observed a tightly connected network mediating *ESR1* and *HIF1A* (Figure 4F) which suggested crosstalk between estrogen and hypoxia. We then compared the expression of Erα across all age groups between females and males and found that Erα expression was slightly greater in females younger than 50 years but decreased after 50 years of age (Figure 4G), which corresponds to the menopausal period. Considering that menopause results in a dramatic increase in cardiovascular disease risk in females (El Khoudary et al., 2020) and that the activation of the cellular response to hormone stimulation is shared by normal aging and AD onset (Figure 4D), we hypothesized that female‐specific hormone alterations during normal aging inhibits the hypoxia response and subsequently contributes to angiogenesis dysfunction (Wu et al., 2024).

### 3.5 Activation of *CREB1* characterizes female AD gene expression

**FIGURE 5:**
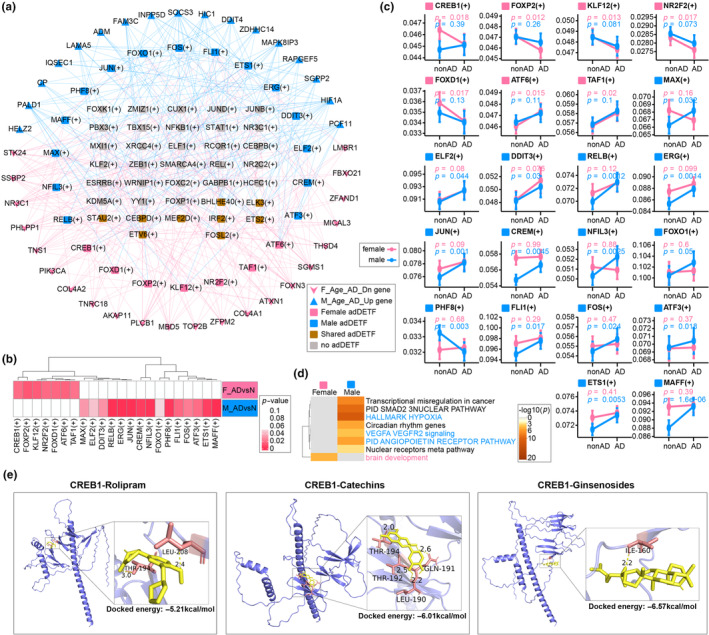
Sex‐specific adDETF analysis. (a) Transcription factor‐target gene regulatory network of female aging Alzheimer's disease (AD) downregulation and male aging AD upregulation. (b) Heatmap of Wilcoxon test *p*‐values for sex‐specific adDETFs. (c) Variations in sex‐specific adDETFs in AD patients and normal controls between males and females. (d) Metascape overrepresentation analysis of sex‐specific adDETFs. (e) Docking diagrams of three drugs bound to CREB1, showing the docking amino acids, hydrogen bond lengths (Å), and binding energies.

Activation of the hypoxia response in males and reduced angiogenesis in females, along with several other sex‐specific activated processes and pathways, including growth factors, hormones, and stress‐induced cellular responses, are involved in normal aging and the onset of female AD. To comprehensively investigate the activated TFs that contribute to these sex‐specific features in AD, PySCENIC was used for TF identification. In total, 129 TFs (Table S10) were identified based on their coexpression and physical binding relationships, with genes related to normal vascular aging and adDEGs shared by both sexes (Figure S8). Among these TFs, 59 were found to regulate the downregulation of female AD aging and the upregulation of male AD aging (Figure 5A). Differential expression analysis of these TFs in AD revealed that *CREB1*(+), *FOXP2*(+), *KLF12*(+), *NR2F2*(+), *FOXD1*(+), *ATF6*(+) and *TAF1*(+) were specifically altered between female AD patients and female controls, while *MAX*(+), *ELF2*(+), *DDIT3*(+), *RELB*(+), *ERG*(+), *JUN*(+), *CREM*(+), *NFIL3*(+), *FOXO1*(+), *PHF8*(+), *FLI1*(+), *FOS*(+), *ATF3*(+), *ETS1*(+) and *MAFF*(+) were specifically altered between male AD patients and male controls (Figure 5A–C). ORA of the TFs indicated that male AD‐specific TFs were enriched in hypoxia (*ATF3*, *DDIT3*, *ETS1*, *FOS*, *JUN*, *NFIL3*, *MAFF*, *RELB*, and *FOXO1*), the VEGF signaling pathway (*ERG*, *ETS1*, *FOXO1*, and *JUN*), and the pid angiopoietin receptor pathway (*ELF2*, *ETS1*, and *FOXO1*) (Figure 5D; Table S11). These findings demonstrated that the activation of hypoxia signaling pathways in male patients with AD is highly dependent on TF activation. Remarkably, *FOS* and *JUN*, which are vital constituents of the hypoxia response (Cummins & Taylor, 2005) and are significant downstream effectors of the cAMP signaling pathway (Figure S9), are highly expressed in male AD patients (Figure 5C), underscoring their importance in sustaining neurodevelopment in the male AD brain (Herdegen et al., 1997; Raivich & Behrens, 2006).

For females, we focused on *CREB1*(+), which participates in VEGFA VEGFR2 signaling (Table S11) and is specifically downregulated in females with AD. Additionally, its target genes, including *PIK3CA* and *PLCB1* are specifically downregulated in female patients with AD (Figure 4D). The protein encoded by *CREB1*(+), a cAMP response element‐binding protein, acts as a downstream effector of the cAMP signaling pathway (Figure S9). It is involved in the regulation of both physiological and pathological cellular processes, affecting neurodevelopment, learning, memory, neuroadaptation, and cell proliferation (Amidfar et al., 2020). In addition, several studies have shown that estrogen can rapidly phosphorylate CREB through a nongenomic mechanism (Szego et al., 2006; Zhou et al., 2005), and that phosphorylated CREB can positively regulate VEGF to promote angiogenesis, affect oxygen perfusion, and consequently impair cognitive function (Burtscher et al., 2021). Therefore, the decrease in the cerebrovascular hypoxic response caused by the decrease in estrogen during aging in females is an important factor influencing the development of AD. Moreover, *FOS* and *JUN* are also regulated by *CREB1* in the cAMP signaling pathway (Figure S9); although they are also upregulated in female AD patients, this increase was not statistically significant. Therefore, their sex‐specific differences could be attributed to sex‐specific differences in *CREB1* expression in AD. This finding also indirectly suggests that the upregulation of *CREB1* in female AD patients is important for the downstream regulation of neurodevelopment by *FOS* and *JUN*. Drugs that target CREB, such as rolipram, have been found to potentially treat AD. Rolipram, a PDE inhibitor, activates CREB, which increases cAMP and cGMP signaling, thereby reversing memory impairment (Monti et al., 2006). Catechins (Li et al., 2009), a flavonoid in green tea, and ginsenoside (Zhao et al., 2009), an active ingredient in ginseng, may also delay memory deficits associated with aging by maintaining the levels of phosphorylated CREB and PKA. We used molecular docking to test whether the three compounds could stably bind to CREB. All three compounds indeed bound to *CREB1* in a stable manner (docked energies < −5 kcal/mol) (Figure 5E) and could be used as potent drugs for the treatment of AD in females.

## 4 DISCUSSION

In our study, we revisited Sun's human cerebrovascular single‐nucleus RNA sequencing data, with a special emphasis on sex differences. We carried out an extensive differential analysis of AD in both sexes, considering a variety of cell types and brain regions. Our findings underscore that the most notable sex‐related disparities in AD are manifested in the endothelial cells of the prefrontal cortical region. The prefrontal cortical region, with its high vascular density and the most distinctive vascular attributes of its endothelial cells, has emerged as an especially pertinent region for investigating cerebrovascular differences between males and females in AD. Our results also reveal the important roles played by other cell types in the sex differences observed in AD. For instance, the number of adDEGs in pericytes showed the greatest difference between sexes (Figure 1F). This finding underscores that in‐depth research on the aging patterns of each specific cell type and their contributions to sex differences in AD is necessary. This will be a major area of focus for future studies.

According to our enrichment of sex‐adDEGs, we concluded that the sex bias in the prevalence and severity of AD may be due to differences in the degree of adaptation of different sexes to hypoxic conditions. In males, we found a greater presence of hypoxia response and angiogenesis pathways, suggesting a stronger defense against BBB damage induced by AD. It is worth noting that angiogenesis is also one of the outcomes of the hypoxic response. When oxygen levels are reduced due to low perfusion in the vasculature after BBB injury in AD patients, the body generates a hypoxic response that promotes angiogenesis to counteract the effects of insufficient oxygen levels. For instance, after hypoxic events, increased *ADM* expression can act either by upregulating the expression of vascular endothelial growth factor or by increasing nitric oxide production in endothelial cells (Ferrero et al., 2018). In male AD patients, an increase in AM suggests the presence of more neuroprotective mechanisms in the male cerebrovascular system. The greater number of hypoxic responses and increased angiogenesis in the endothelial cells of the vasculature in male AD patients could be reasons for the milder disease symptoms in males.

In female AD patients, there is a reduction in vascular development and an increase in the presence of antigens, which might be due to the substantial decrease in hormone levels after menopause in females. The sex differences in adaptation to hypoxic conditions have been proven by many studies to be related to hormone levels (Kazi & Koos, 2007; Snyder et al., 2018). Estrogens have a protective effect against hypoxia. They can enhance the expression of hypoxia‐inducible factor 1, thereby helping cells adapt to a low‐oxygen environment. After menopause in females, the level of estrogen in the body decreases significantly, weakening the ability to adapt to hypoxic conditions and facilitating BBB damage leading to AD. It has also been shown that estrogen is beneficial for improving memory in humans and mice (Resnick et al., 1997; Xu & Zhang, 2006), further emphasizing the important role of hormones. Therefore, a deeper analysis revealed that differences in hormone levels between elderly males and females lead to differences in the degree of adaptation to hypoxic conditions. This may also explain why females have a greater incidence and severity of disease than males. On the other hand, while antigen presentation was also observed in males during subsequent baseline comparisons, the *HLA‐C* gene remained highly expressed in females. This sex‐specific expression of certain genes, such as *HLA‐C*, warrants further investigation. Moreover, we found that sex differences in AD manifest at an early stage of the disease. This suggests that our sex‐specific treatment approaches for AD need to intervene at an early stage of neurodegeneration.

We then validated the sex‐adDEGs, sex‐differing pathway genes, and single genes using independent datasets (Lau et al., 2020; Yang et al., 2022), which all yielded robust validation results (Figure 2C; Figure S2C). However, the antigen presentation, angiogenesis, and PDGF pathways were not validated in females. This might be due to the limited sample size. Specifically, the single‐cell data for female AD patients in the prefrontal cortex region in this dataset were provided by only one female AD patient (Yang et al., 2022). Despite this limitation, differences in the expression of genes such as *HLA‐C*, *PTK2*, and *COL4A2* between males and females remain to be validated. We also observed differences between males and females in terms of other types of vascular cells in AD, but the phenomenon of hypoxic response pathways being more enriched in males was not observed in smooth muscle cells. This may be due to the smaller number of smooth muscle cells (Figure 1E).

To study the impact of vascular aging on the sex bias in AD, we defined our own set of vascular aging genes, given that the aging datasets (Aging Atlas, CSgene, cellage) are not independent sets of vascular tissue aging genes and that the effects of sex have not been considered. The vascular aging genes we described have considerable overlap with the aging datasets, indicating that there is a certain similarity between vascular aging and body aging. In addition, we acknowledge that our identified genes were related to cerebrovascular aging. However, considering the similarity of vascular function across different parts of the human body and that accelerated artery aging is associated with the early development of AD (Oh et al., 2023), we believe it is reasonable to use aging data from the aorta and coronary arteries as a surrogate for cerebrovascular aging. There seems to be no functional difference in vascular aging genes between the sexes. However, there are male–female differences in the genes responsible for vascular aging that lead to the development of AD. This finding suggests that the study of male–female differences in AD should be analyzed at a more specific and detailed level, which may explain why sex differences have not been observed in many AD studies. In our study, we concluded that the differential adaptation to hypoxic conditions between males and females contributes to the sex‐biased pathogenesis of AD. During the normal aging process, the oxygen content in the brain decreases (Burtscher et al., 2021). This reduction is further exacerbated in AD patients due to damage to the BBB, leading to an even greater decrease in the oxygen content of the brain. In response to this hypoxic environment, males exhibit a heightened hypoxic response triggered by certain genes (*ADM*, *INPP5D*, *SOCS3*, *HIF1A*, *LAMA5*, *DDIT4*, *CP*, and *SGPP2*). This response helps combat the onset of aging and AD. Conversely, in females, the downregulation of genes related to vascular development (*ZFPM2*, *NR3C1*, *COL4A2*, *MBD5*, *PLCB1*, and *PIK3CA*) results in a deficiency of blood vessels, which results in a lack of adequate cerebral vasculature to maintain normal brain responses and a greater degree of pathology and greater susceptibility to pathology in female patients with AD.

Our analysis of TFs confirmed sex differences in AD, and we identified *CREB1* as a potential target for female‐specific therapy. Moreover, experiments have shown that female mice deficient in the *CREB* gene are more likely to develop cognitive impairment with age than their male counterparts (Hebda‐Bauer et al., 2007), validating our results. It remains unclear that the efficiency of the CREB mediated therapeutic interventions in the context of AD. In our follow‐up work, we would like to examine the effectiveness of particular small compounds in treating AD mice, especially female mice, and confirm whether the compounds' affinity was highly dependent on CREB1.Therefore, the approach of this paper, which is to study the sex‐biased pathogenesis of AD from the perspective of different patterns of vascular aging, is feasible. The vascular differences in normal aging between males and females are one of the causes of the sex bias in AD.

Although females have a longer average lifespan than males, which leads to a greater prevalence of AD in females, longevity cannot fully explain the greater frequency and lifetime risk in females. Moreover, an important fact that cannot be overlooked is that the occurrence of menopause in females results in differences in hormone level changes during aging between sexes, which significantly affect their different physiological responses, this will be a key focus of future research. As the global trend of aging intensifies, the proportion of people with AD will inevitably increase, and there is an urgent need for precision treatment of AD, especially considering sex factors. Overall, by exploring the sex‐biased pathogenesis of AD from the perspective of vascular aging in both sexes, we have provided new insights for the study of sex bias in AD and have made significant contributions to guiding sex‐specific precision treatment of AD.

## 5 CONCLUSIONS

By analyzing the largest brain vasculature single‐nucleus RNA sequencing data at single‐cell resolution, we were able to uncover critical sex‐specific differences in the etiology of AD. We observed that male AD patients display enhanced angiogenesis and hypoxia responses, whereas, female AD patients demonstrate impeded vascular growth and development, with these differences developing in the early stages of the illness. We also identified genes related to vascular aging in both healthy males and females, and further highlighted that normal vascular aging may contribute to the observed sex differences in AD patients. In addition, we examined sex‐specific TFs that may explain these differences. Our findings highlight the importance of considering sex‐specific characteristics when developing customized treatment options for Alzheimer's patients.

## AUTHOR CONTRIBUTIONS

KL, DW, XB, and BW came up with the design and conception. Development of methodology was performed by PL, DW, and XB. Acquisition, analysis and interpretation of data were performed by PL, DW, XB, DX, YX, JC, YS, JW, JCh, and LY. YM conducted the mouse experiment. Writing, review, and/or revision of the manuscript were performed by PL, DW, XB, DX, YM, BW, and KL. PL, XB, DX, and YM contributed equally to this study and shared co‐first authors. All authors read and approved the final manuscript.

## FUNDING INFORMATION

This work was supported by Hainan Provincial Natural Science Foundation of China (823RC581 and 822QN462), the Program of Hainan Association for Science and Technology Plans to Youth R & D Innovation (QCQTXM202212), Major Science and Technology Program of Hainan Province (ZDKJ2021040), the National Natural Science Foundation of China (32160152), Innovative Research Project for Graduate Students in Hainan Province (Qhys2021‐354), Innovative Research Project for Graduate Students in Hainan Medical University (HYYS2021B08), Bioinformatics for Major Diseases Science Innovation Group of Hainan Medical University.

## CONFLICT OF INTEREST STATEMENT

The authors declare no competing interests.

## Supporting information


Figure S1.

Table S1.

## Data Availability

The main data for this study come from the research conducted by Sun et al. (2023), with count matrices and metadata for all analyzed cells available at http://compbio.mit.edu/scADbbb/. All codes are available at https://github.com/DBprojects‐lab/adCVaging.
