# Supplementary material for: Activation of the hypoxia response in the aging cerebrovasculature protects males against cognitive impairment
Source: Aging Cell. 2024 Jul 2;23(10):e14264. doi: 10.1111/acel.14264 (PMC11464119; doi:10.1111/acel.14264)
Supplement: Supplementary file 1 — Figure S1. [file ACEL-23-e14264-s002.docx]

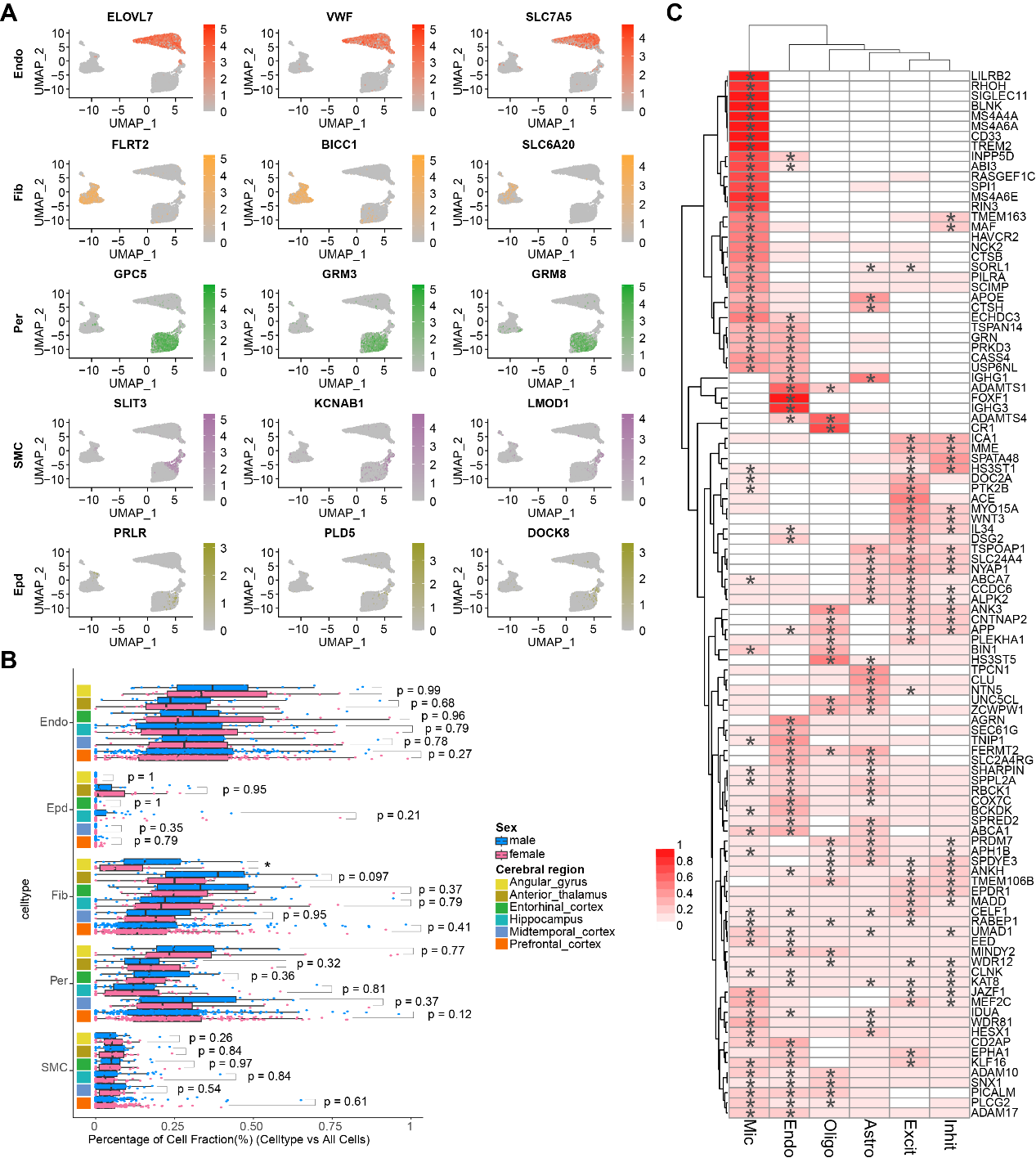


Fig. S1: Analysis of brain vascular cell types in sex-stratified samples across six regions and AD-GWAS risk gene enrichment. (A) UMAP plot of gene markers for brain vascular cell types. (B) Sex-stratified vascular cell type proportion comparison across six regions (*: p<0.05). (C) Expression Weighted Cell Type Enrichment (EWCE) of AD-GWAS Risk Genes. Asterisk (*: Enrichment scores greater than the average (1/6)).


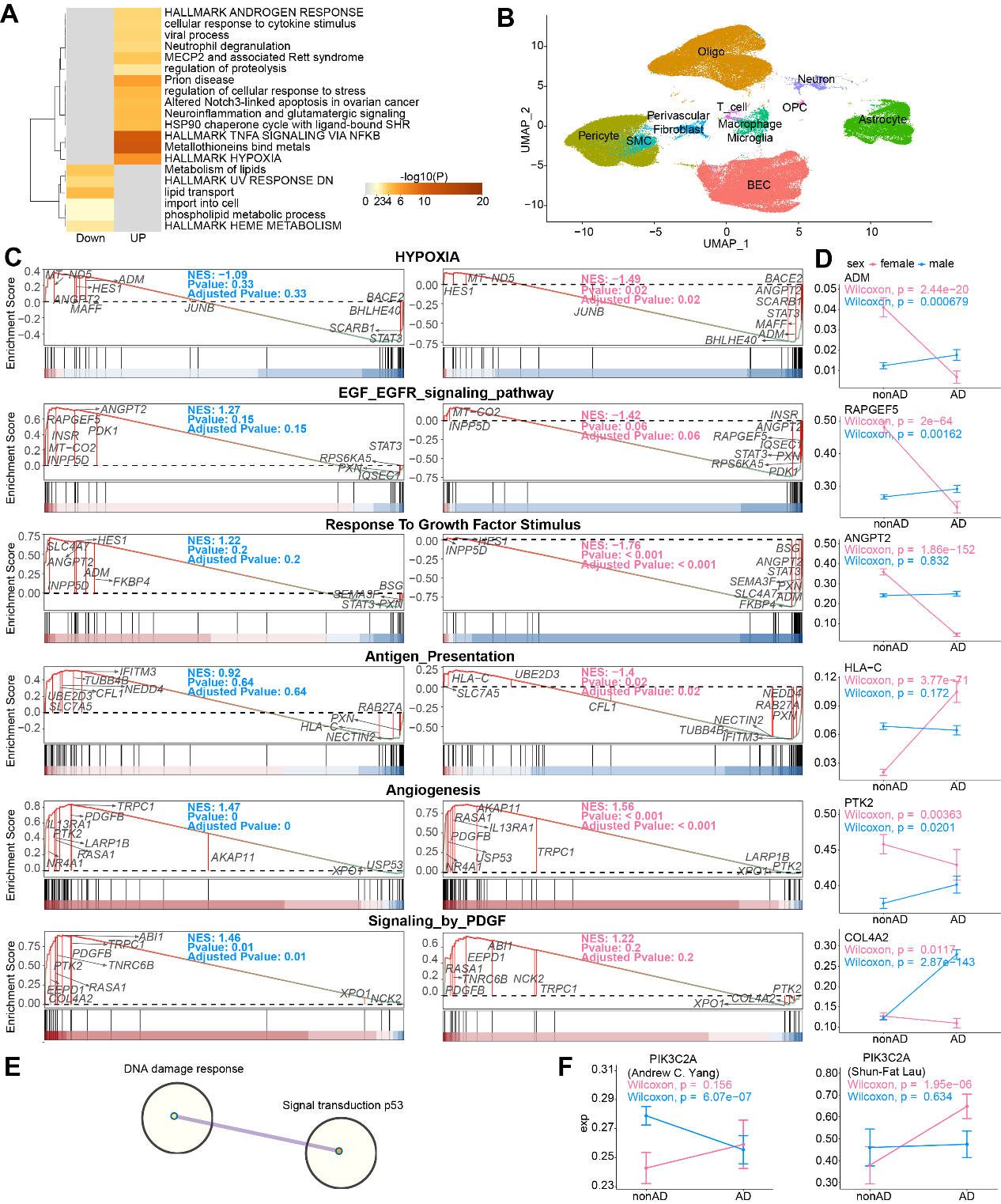


Fig. S2: Over-representation analysis of overlapping genes between male AD and female AD, and independent dataset validation of sex differences. (A) Over-representation analysis of co-downregulated and co-upregulated adDEGs in both sexes. (B) UMAP plot of Andrew C. Yang's single-cell brain vascular data. (C) The GSEA validation of differential pathways in male (left) and female (right) using Andrew C. Yang's single-cell brain vascular data, with the x-axis representing the ranking of ADlog2FC values from high to low. (D) The sex-specific changes in key genes in AD. (E) Over-representation analysis of 10 genes decreased in female AD and increased in male AD. (F) Validation of the PIK3C2A gene in Andrew C. Yang (left) and Shun-Fat Lau (right).


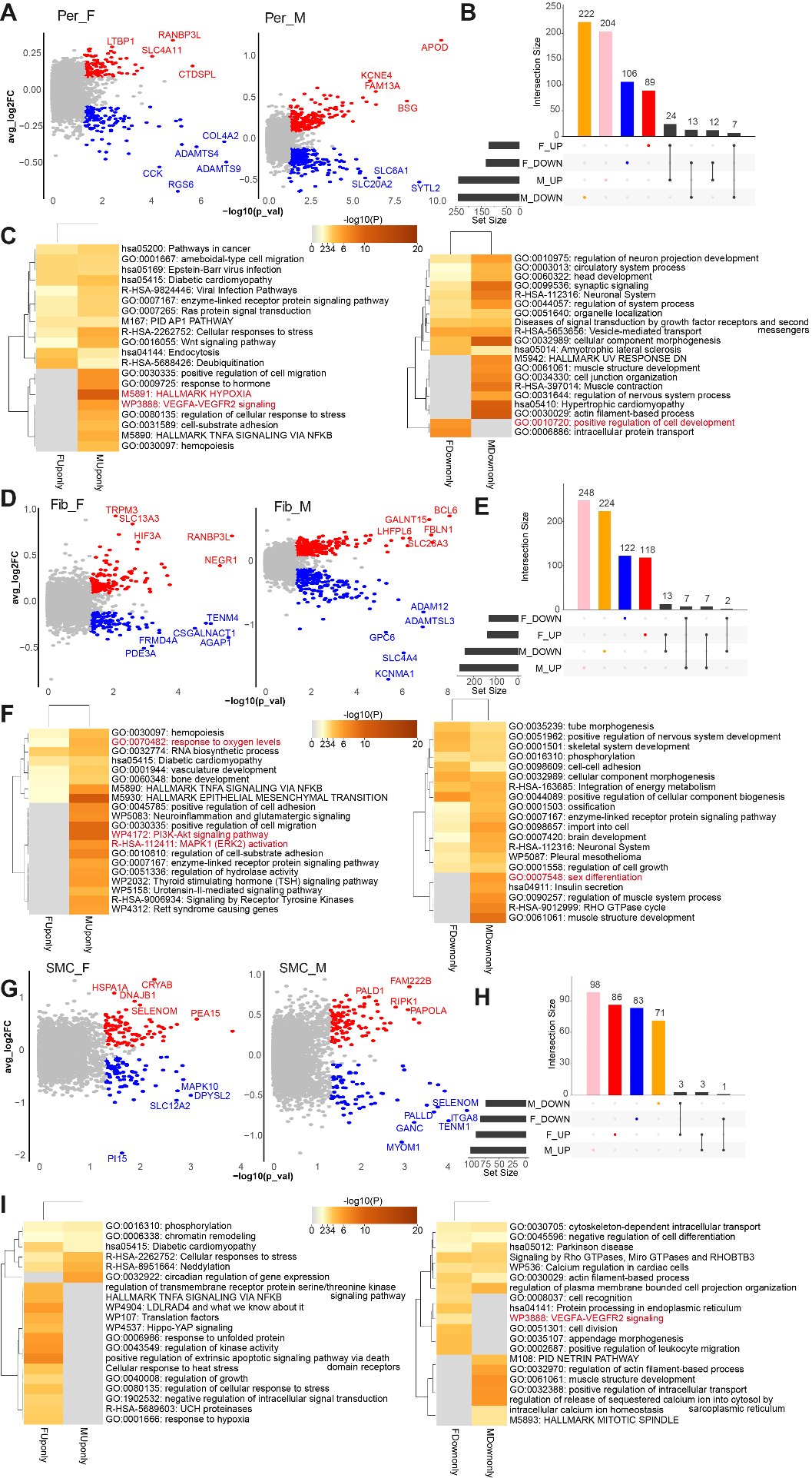


Fig. S3: Volcano plots of both sexes adDGEs for three other types of vascular cells; Upset plots of upregulated and downregulated genes in males and females; Over-representation analysis of sex-adDEGs. A-C: Pericytes; D-F: Fibroblasts; G-I: Smooth Muscle Cells.


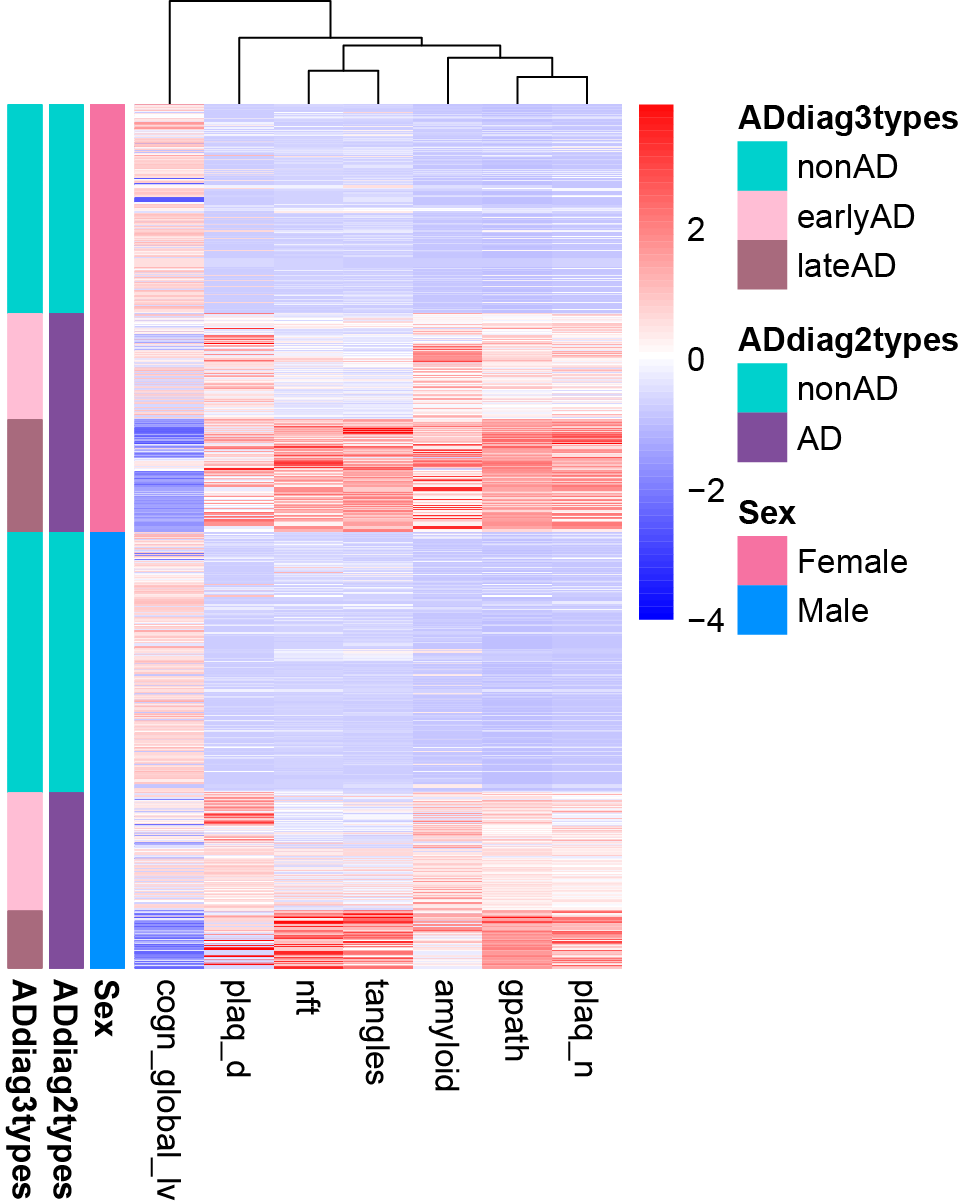


Fig S4: Sample distribution of six clinico-pathological traits in conjunction with cognitive impairment.


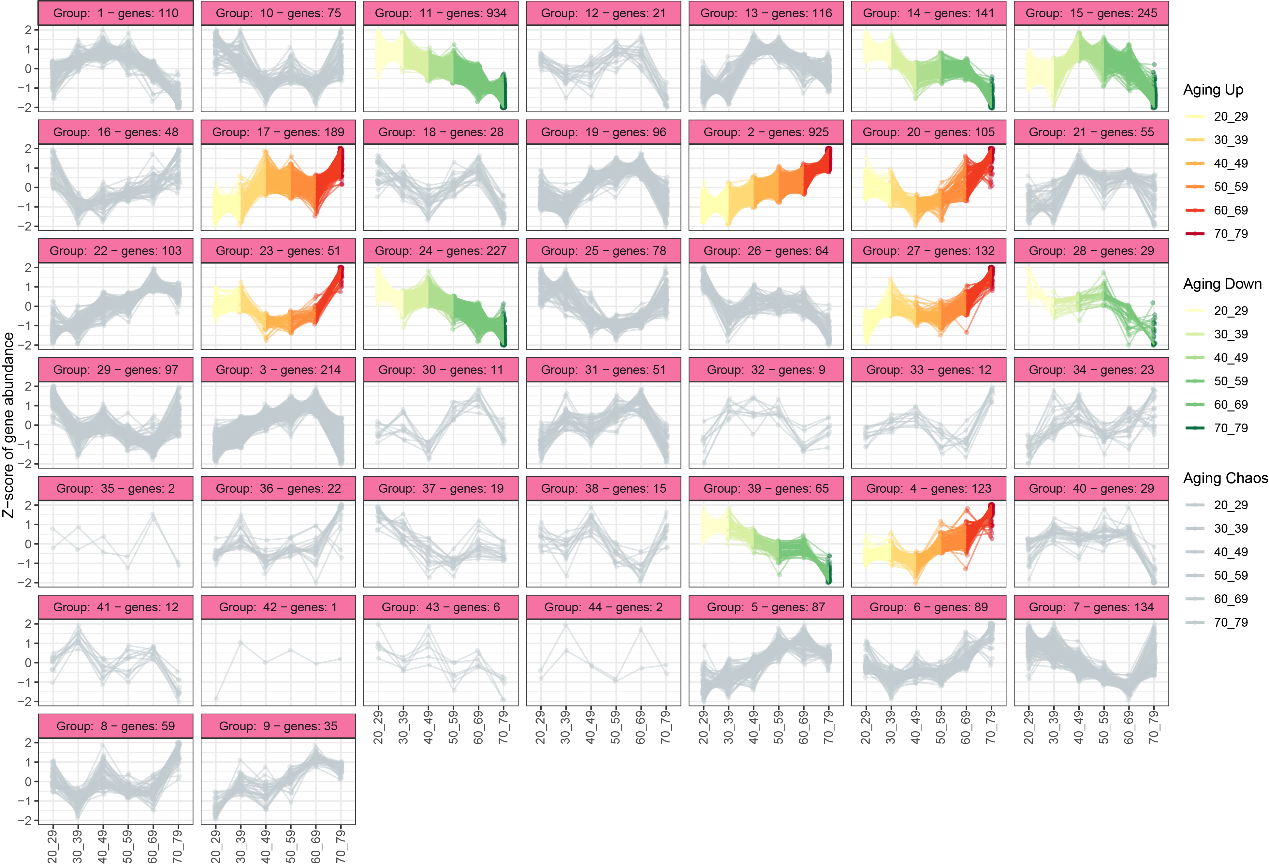


Fig S5: DegPatterns clustering group diagram of aging-associated DEGs in normal vessels for females.


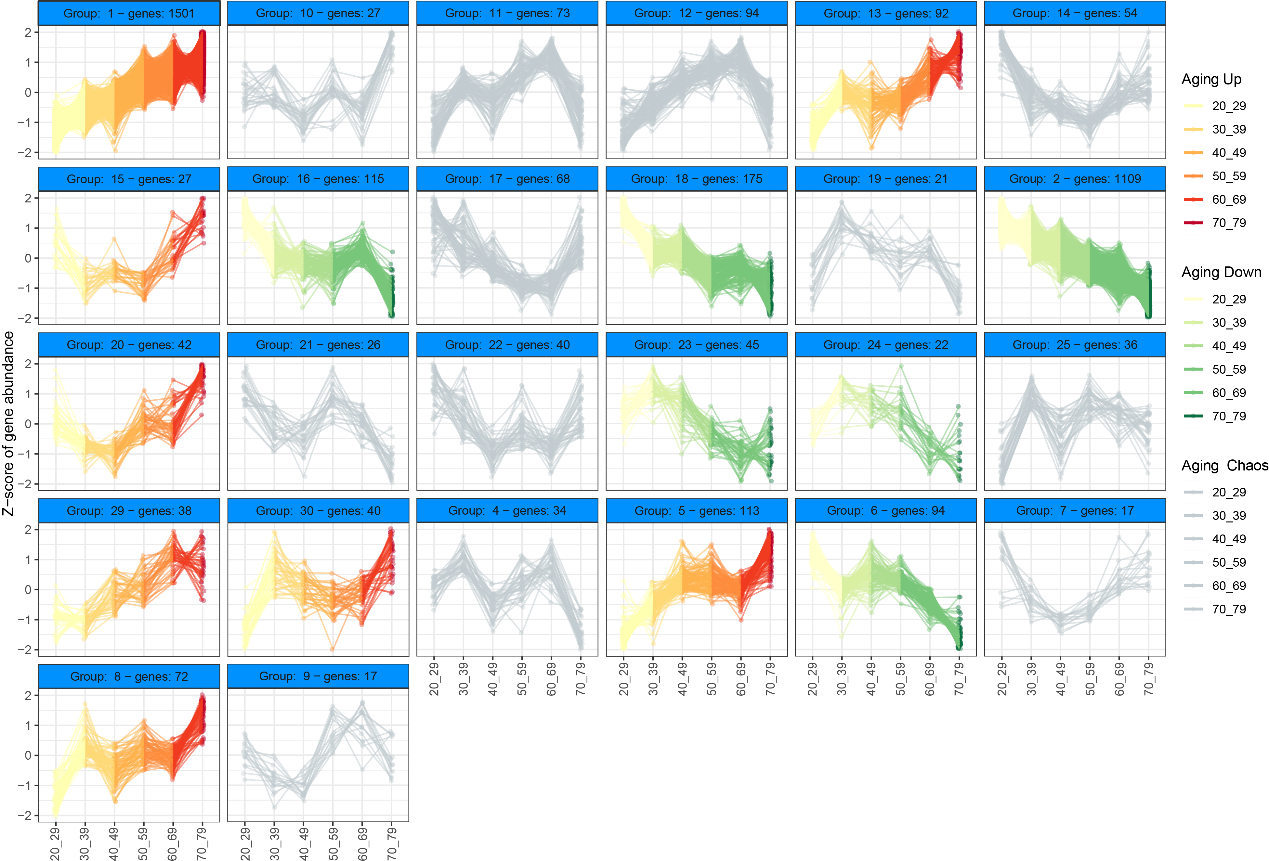


Fig S6: DegPatterns clustering group diagram of aging-associated DEGs in normal vessels for males.


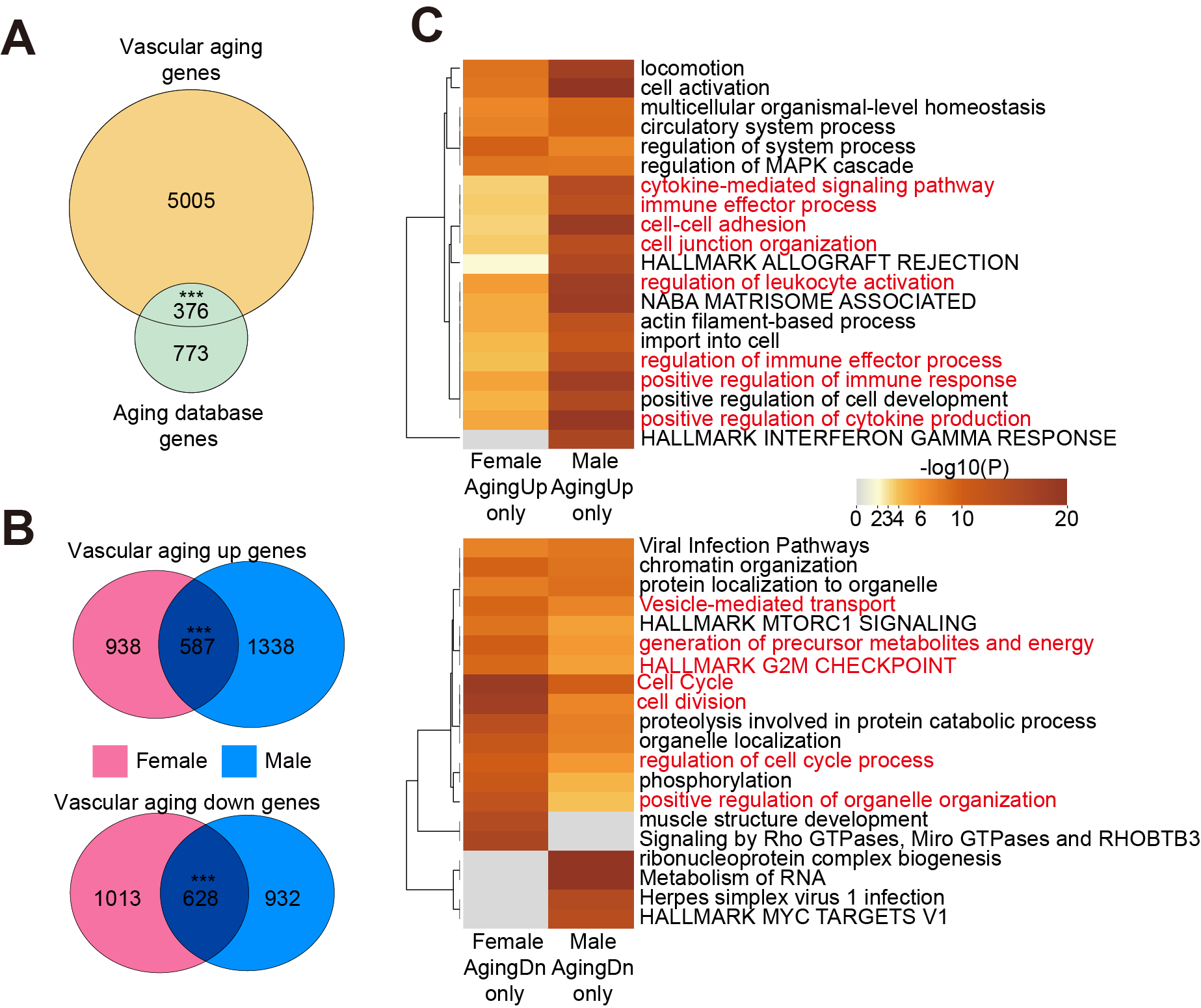


Fig. S7: Vascular normal aging gene overlap and sex-specific aging genes over-representation analysis. (A) Overlapping Venn diagram of vascular aging genes with aging datasets (Aging Atlas, CSgene, cellage). (B) Overlapping Venn diagram of upregulated and downregulated vascular aging genes in males and females (***: p<0.001). (C): Over-representation analysis of sex-specific upregulated and downregulated vascular aging genes.


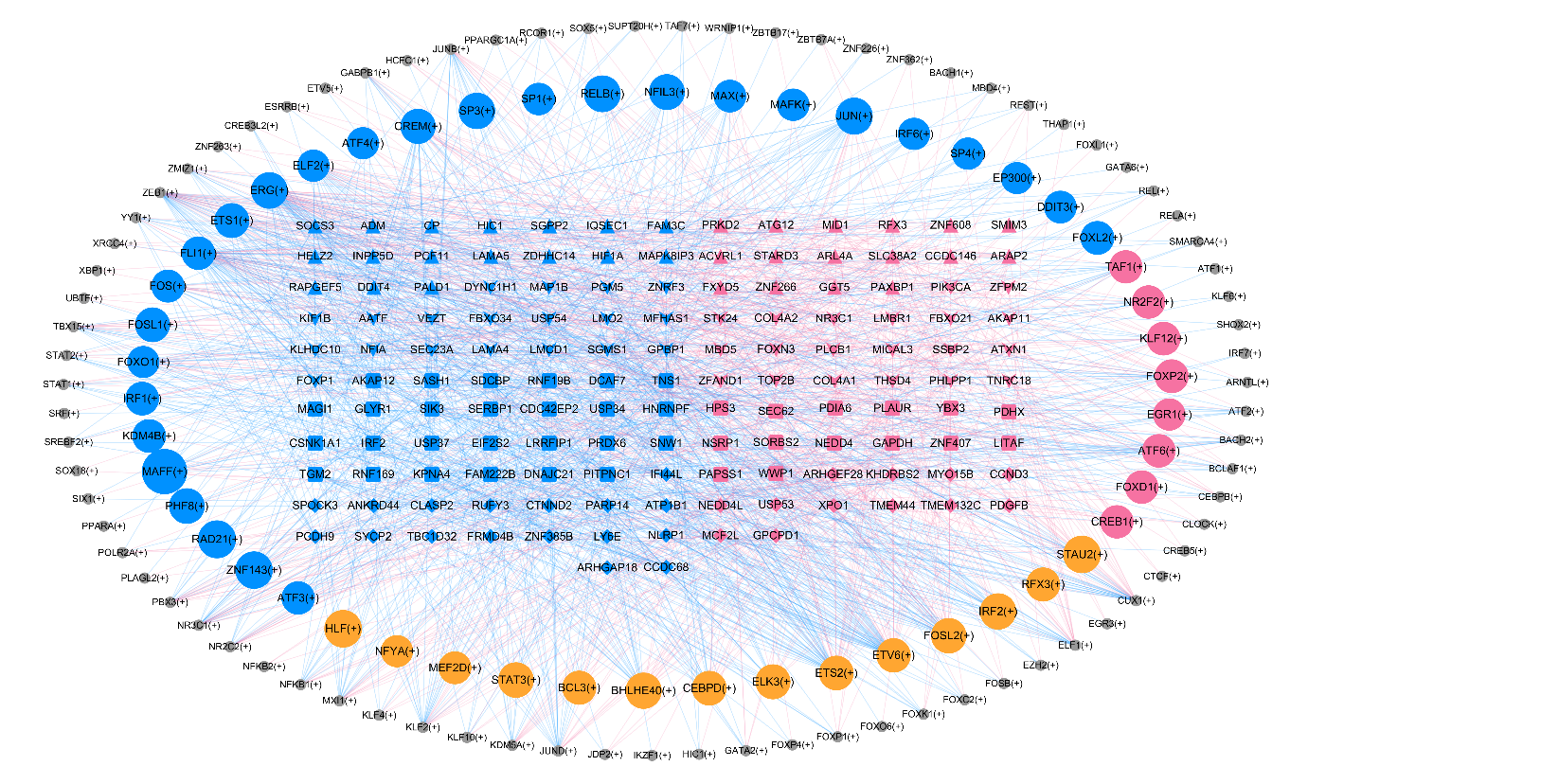


Fig S8: Transcription factor-target gene regulatory network of normal vascular aging and AD-associated differentially expressed genes (adDEGs).


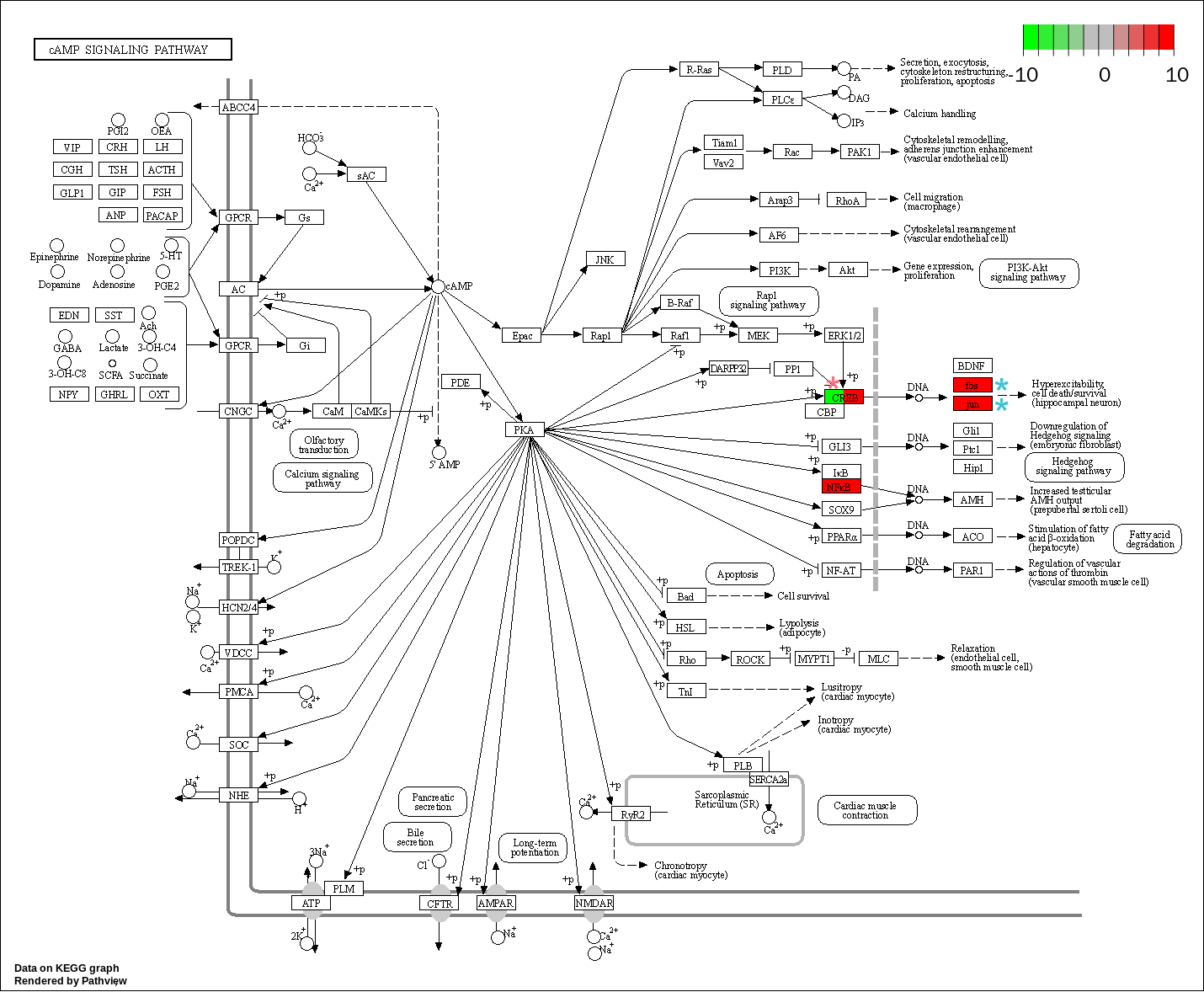


Fig. S9: Schematic of the KEGG-cAMP signaling pathway. The left side of the box represents females, the right side represents males, and the color represents the log2FC (log2 Fold Change) value. *: p<0.05.
